# Chickpea flour (*Cicer arietinum* L) improves viability of potentially probiotics *Lactobacillus plantarum* S180.7 and *Lactobacillus plantarum* SBR64.7 in milk-based formulations

**DOI:** 10.1371/journal.pone.0362095

**Published:** 2026-11-02

**Authors:** Larissa dos Santos Freitas, Sandra Fernandes Arruda, Maria Carolina Mesquita, Thalyson Guirra Eneias, Marcio Antônio Mendonça

**Affiliations:** 1 Post-Graduate Program in Animal Sciences, College of Agronomy and Veterinary Medicine, Campus Universitário Darcy Ribeiro, University of Brasília, Brasília, Brazil; 2 Department of Nutrition, College of Health Sciences, University of Brasília, Brasília, Federal District, Brazil; 3 College of Agronomy and Veterinary Medicine, University of Brasília, Brasília, Federal District, Brazil; SRMIST (Deemed to be University): SRM Institute of Science and Technology (Deemed to be University), INDIA

## Abstract

This study evaluated the effect of adding chickpea flour to milk-based formulations on physicochemical parameters, the survival, and viability of potentially probiotic microorganisms. Four formulations were prepared using skimmed milk (10%) and sucrose (10%), each containing *Lactobacillus plantarum* S180.7 (CF1) or *L. plantarum* SBR64.7 (CF2), with or without 4.5% chickpea flour (TF1 and TF2). Composition, physicochemical properties, total phenolic content, antioxidant activity, and bacterial viability were assessed during 24 hours of fermentation and 28 days of storage at 4°C. Formulations with chickpea flour exhibited a greater pH decrease (ΔpH, 24–0 h: TF1 = –1.81 and TF2 = –1.38) than controls (CF1 = –0.58 and CF2 = –0.52), along with higher titratable acidity after 6 and 9 hours, respectively. Chickpea flour enhanced bacterial growth, reaching 8 Log CFU/mL, and increased phenolic content without affecting antioxidant potential. Viability remained stable and higher than controls, confirming improved probiotic stability in milk-based formulations.

## 1. Introduction

Lactic acid bacteria (LABs) play a crucial role in the development of fermented products due to their ability to ferment sugars, primarily producing lactic acid [1]. LABs are widely employed in industrial processes, as the diverse compounds generated during their fermentative metabolism enhance the sensory and nutritional properties of foods [2]. The probiotic effects of LABs against pathogenic bacteria colonizing the gastrointestinal tracts also explain their broad application in fermented foods intended for human consumption [3].

The rising worldwide interest in “functional foods” with probiotic lactic acid bacteria has stimulated researches for isolation and identification of new LAB from natural sources. Recently, Leandro *et al*. [4] isolated two potentially probiotic strains, *Lactobacillus plantarum* S180.7 and *Lactobacillus plantarum* SBR64.7, from the relatively unexplored sources of artisanal salami and grass silage, respectively. The authors demonstrated that these strains are promising probiotic candidates, as they exhibited high viability when exposed to in vitro gastric and pancreatic juice digestion. A key criterion for evaluating probiotic bacteria is their survival under adverse gastrointestinal tract conditions, which ensures their viability and functional capacity in the intestine. During transit, these bacteria must withstand challenges such as stomach acidity and pepsin activity. In this context, the food matrix plays a protective role, acting as a physicochemical shield and increasing bacterial tolerance against digestive stress [5].

The species *Lactobacillus plantarum* holds “generally recognized as safe” status (GRAS) and is noted for its strong ability to survive under the adverse conditions of the gastrointestinal tract, as well as, its ability to adhere to epithelial cells in both humans and animals [6].

Milk is considered a suitable matrix for the growth of lactic acid bacteria. However, the literature generally reports that *Lactobacillus plantarum* has limited application in milk processing due to its low proteolytic activity, which makes it dependent on additional free amino acids, peptides, vitamins, minerals, and bioactive compounds to sustain growth in milk alone [7–9].

Chickpea (*Cicer arietinum L*.) flour, one of the most widely consumed legumes, is considered a rich source of proteins (47.4–55.8%), essential amino acids (leucine 7.6, isoleucine 4.8, lysine 6.0, phenylalanine 5.6, tyrosine 3.6, threonine 3.9, and valine 5.6 g/100 g protein), fiber (3.9–11.2%), and minerals (2.8–3.2%). It also contains high concentrations of monosaccharides (0.32–0.97 g/100 g), disaccharides (sucrose 1.09–2.28 g/100 g), and oligosaccharides (3.87–6.98 g/100 g) [10,11]. Therefore, chickpea flour represents a promising source of nutritional and bioactive compounds for *L. plantarum* S180.7 and *L. plantarum* SBR64.7 when added into milk, potentially enhancing bacterial viability and survival.

The present study evaluated the effect of incorporating chickpea flour into milk-based formulations on physicochemical parameters, in addition to the survival and viability of the potentially probiotic strains *L. plantarum* S180.7 and *L. plantarum* SBR64.7 during fermentation, storage, and simulated in vitro gastrointestinal conditions.

## 2. Materials and methods

### 2.1. *L. plantarum* S180.7 and *L. plantarum* SBR64.7 stock culture

Stock cultures of *L. plantarum* S180.7 and *L. plantarum* SBR64.7, obtained from the culture collection of the Food Hygiene Laboratory, Department of Nutrition, School of Health Sciences, University of Brasília, Federal District, Brazil, were used in this study. Cultures were activated by inoculating 100 µL of the frozen stock into De Man Rogosa Sharpe (MRS) broth (Acumedia-Neogen, Michigan, USA) followed by incubation at 37 °C for 24 h in a bacteriological incubator (Ethik Technology, Vargem Grande, SP). The cultures were subsequently resuspended in MRS broth containing 20% glycerol and stored at −80 °C for later analyses [12].

#### 2.1.1. Preparation of *L. plantarum* S180.7 and *L. plantarum* SBR64.7 cultures.

Stock cultures of *L. plantarum* S180.7 and *L. plantarum* SBR64.7 were used in this study. Briefly, 100 μL of each stock culture was inoculated into 5 mL of De Man Rogosa Sharpe (MRS) broth (Acumedia-Neogen, Michigan, USA) and activated by incubation at 37 °C for 24 h in a bacteriological incubator (Ethik Technology, Vargem Grande, SP). Subsequently, 200 μL of each activated culture (2% v/v inoculum) was transferred to 10 mL of fresh MRS broth and incubated at 37 °C for an additional 24 h. The cultures were then centrifuged at 2,655 × g for 5 min at 4 °C, and the resulting cell pellets were washed twice with 0.85% saline solution. Cell suspensions were adjusted to 6 × 10^8^ CFU/mL according to the McFarland scale, and 1% of this inoculum was added to 100 mL of each milk-based formulation.

### 2.2. Formulation

Four milk-based formulations were prepared, containing either *L. plantarum* S180.7 or *L. plantarum* SBR64.7, with or without 4.5% chickpea flour. The formulations were as follows:

- Control Formulation 1 (CF1): reconstituted skimmed milk (10%) + sucrose (10%) + *L. plantarum* S180.7 (8 Log CFU);
- Test Formulation 1 (TF1): reconstituted skimmed milk (10%) + sucrose (10%) + *L. plantarum* S180.7 (8 Log CFU) + chickpea flour (4.5%);
- Control Formulation 2 (CF2): reconstituted skimmed milk (10%) + sucrose (10%) + *L. plantarum* SBR64.7 (8 Log CFU);
- Test Formulation 2 (TF2): reconstituted skimmed milk (10%) + sucrose (10%) + *L. plantarum* SBR64.7 (8 Log CFU) + chickpea flour (4.5%).

All formulations were homogenized using a blender (Walita, model RI7630/22) at power level 1 for 30 s, autoclaved at 121 °C for 20 min, and stored at 4 °C for 16 h. The formulations were then inoculated with the respective cultures and incubated in a bacteriological oven at 37 °C for 24 h.

### 2.3. Characterization of the chemical composition of the formulations

The moisture content and the concentrations of proteins, lipids, and ashes were determined according to standard analytical procedures. Moisture was analyzed following the Analytical Standards of the Adolfo Lutz Institute [13]. Protein content was measured using the Kjeldahl method 991.22 [14]. Lipids were extracted using method Am 5−04 with a lipid extractor (model ANKOM XT15, Ankom Technology, New York, USA) [15]. Ash content was determined by method 945.45 [14]. The concentration of total carbohydrates was estimated by difference, calculated as 100% minus the sum of moisture, protein, lipid, and ash contents, following method 986.25 [14].

### 2.4. Growth kinetics

The physicochemical characteristics and viability of L. *plantarum* S180.7 and *L. plantarum* SBR64.7 were analyzed at time intervals of 0, 3, 6, 9, 12 and 24 h of fermentation.

#### 2.4.1. pH, titratable acidity, and total soluble solids (TSS).

The pH of the formulations was measured using a digital pH meter (PHS-3E, Birigui, Araucária, PR, Brazil), according to the Analytical Standards of the Adolfo Lutz Institute [13]. Titratable acidity was determined by homogenizing 10 mL of each formulation in 100 mL of deionized water, followed by titration with 0.1 mol/L NaOH, using phenolphthalein as an indicator [13]. Total soluble solids were quantified with a digital refractometer (ATAGO®, model 1T) following method 932.12 [14], and results were expressed in °Brix. All analyses were performed in triplicate.

#### 2.4.2. Viability.

The viability of the *L. plantarum* S180.7 and *L. plantarum* SBR64.7 strains was determined by the *Drop plate* technique. Serial dilutions were carried out in saline solution, and the diluted samples were plated onto Petri dishes containing MRS agar. The plates were incubated at 37°C for 48 h and the viability was determined by the Colony Forming Units (CFU) counting [16]. The results were expressed as log CFU mL^-1^.

#### 2.4.3. Total phenolic concentration and antioxidant potential of formulations.

The total phenolic concentration and antioxidant potential of the formulations were evaluated at 0, 3, 6, 9, 12, and 24 h of fermentation with *L. plantarum* S180.7 and *L. plantarum* SBR64.7.

2.4.3.1 Sample clarification: Sample clarification was performed according to the method described by Carrez [17] and Acker *et al*. [18]. Briefly, 10 μL of Carrez I solution (15% K_4_Fe(CN)_6_·3H_2_O) and 10 μL of Carrez II solution (30% ZnSO_4_·7H_2_O) were added to 500 μL of each formulation. The final reaction volume was adjusted to 1 mL with deionized water. After homogenization, the reaction was centrifuged at 7,800 × g for 15 min at 5 °C. The supernatant was collected and stored at −80 °C until analysis.

2.4.3.2 Total phenolic content: Total phenolic content was determined using the Folin–Ciocalteu assay, as described by Singleton and Rossi [19]. Briefly, 20 μL of clarified sample, 100 μL of Folin–Ciocalteu reagent (1:10, v/v), and 80 μL of 7.5% Na_2_CO_3_ were added to a microplate. The reaction mixture was incubated for 60 min at room temperature under light protection, and absorbance was measured at 765 nm using a spectrofluorometer (SpectraMax M Series Multi-Mode Microplate Readers, San Jose, CA, USA). A gallic acid calibration curve was constructed at concentrations ranging from 0 to 1,000 μmol/L, and results were expressed as μmol gallic acid/L of formulation.

2.4.3.3 Ferric reducing antioxidant power (FRAP) assay: The antioxidant potential of the formulations was determined using the FRAP assay, as described by Benzie and Strain [20]. Briefly, 4 μL of deionized water, 20 μL of clarified sample, and 180 μL of FRAP reagent were added to a microplate, and absorbance was monitored at 593 nm every 30 s for 10 min using a spectrofluorometer (SpectraMax M Series Multi-Mode Microplate Readers, San Jose, CA, USA). A calibration curve was prepared with hydroxy-2,5,7,8-tetramethylchromane-2-carboxylic acid (Trolox, Sigma-Aldrich, St. Louis, MO, USA) at concentrations ranging from 0 to 1,500 μmol/L. Results were expressed as μmol Trolox equivalents (TE)/L of formulation.

2.4.3.4 DPPH● (2,2-diphenyl-1-picrylhydrazyl) scavenging capacity assay: The antioxidant capacity of the formulations was determined using the DPPH● assay, as described by Cheng *et al*. [21]. Briefly, five different volumes of clarified sample (5, 10, 20, 30, and 40 μL) were added to separate wells of a microplate, and the final volume was adjusted to 100 μL with deionized water. Subsequently, 100 μL of 0.208 mmol/L DPPH● solution was added to each well, and absorbance was monitored at 515 nm for 30 min using a spectrofluorometer (SpectraMax M Series Multi-Mode Microplate Readers, San Jose, CA, USA). For each sample, a curve was constructed by plotting % DPPH● scavenged against sample volume, and the radical scavenging capacity (RDSC) was expressed as μmol Trolox equivalents (TE)/L of formulation.

### 2.5. Physicochemical characterization and viability of *L. plantarum* S180.7 and *L. plantarum* SBR64.7 strains in the formulations during stored for 28 days at 4ºC

The physicochemical parameters (pH, titratable acidity, and total soluble solids) and the viability of *L. plantarum* S180.7 and *L. plantarum* SBR64.7 in the formulations were evaluated after 0, 7, 14, 21, and 28 days of storage at 4 °C, as described in sections 2.4.1 and 2.4.2. The total phenolic content and antioxidant potential of the formulations were determined at 0 and 28 days of storage at 4 °C, following the procedures described in section 2.4.3.

#### 2.5.1. Visual stability.

The visual stability of all formulations was evaluated at 0 and 28 days of storage at 4 °C. Briefly, 5 mL of each formulation were transferred into glass tubes and stored at 4 °C. At each time point (0 and 28 days), the height of the supernatant was measured using a ruler to determine phase separation (supernatant and pellet). Phase separation was quantified as the height of the supernatant (mm) and converted into milliliters, as described by Zaaboul *et al*. [22].

#### 2.5.2. Water-holding capacity.

The water-holding capacity (WHC) of the formulations was determined according to Guzmán-González *et al*. [23], with modifications. Briefly, 25 mL of each formulation were centrifuged at 500 × g for 10 min at room temperature, and the supernatant was carefully removed and weighed. WHC was calculated using the following Equation 1:


$$\begin{array}{c} \text{WHC}=\left(\text{1}-\frac{{\text{m}}_{\text{s}}}{{\text{m}}_{\text{y}}}\right)*\text{100}\% \end{array}$$ (1)

Where:

- WHC = water-holding capacity (%);
- ms = mass of supernatant after centrifugation (g);
- my = mass of formulation before centrifugation (g).

### 2.6. Survival of *L. plantarum* S180.7 and *L. plantarum* SBR64.7 under *in vitro* gastrointestinal simulated digestion

The viability and survival of *L. plantarum* S180.7 and *L. plantarum* SBR64.7 in formulations fermented for 24 h were evaluated under three different in vitro gastrointestinal simulated digestion conditions: (i) exposure to artificial gastric juice, (ii) exposure to artificial intestinal juice, and (iii) sequential exposure to both gastric and intestinal juices.

#### 2.6.1. Gastric juice simulated digestion.

The analyses were performed as described by Bautista-Gallego *et al*. [24], with minor modifications. Gastric juice was prepared using 0.205 g/L NaCl, 0.06 g/L KH_2_PO_4_, and 0.037 g/L KCl, with the pH adjusted to 2.0, followed by autoclaving at 121 °C for 15 min. After sterilization, pepsin (0.0133 g/L; Dinâmica®, Indaiatuba-SP, Brazil) was added to the gastric juice. For the gastric digestion simulation, a 1:1 formulation-to-gastric juice solution was prepared and incubated at 37 °C for 90 min, according to Calabuig-Jiménez *et al*. [25]. The digested samples were then centrifuged at 7,871 × g at 4 °C for 5 min, and the pellet was washed twice with saline and centrifuged under the same conditions. Viability of the strains was determined using the drop plate technique, as previously described, and results were expressed as log CFU/mL of formulation.

#### 2.6.2 Intestinal juice simulated digestion.

The analyses were performed as described by Bautista-Gallego *et al*. [24], with minor modifications. Intestinal juice was prepared using 50.81 g/L Na_2_HPO_4_, 8.50 g/L NaCl, and 3.00 g/L bile salts, with the pH adjusted to 8.0, followed by autoclaving at 121 °C for 15 min. After sterilization, pancreatin (0.1 g/L; Sigma-Aldrich, St. Louis, MO, USA) was added to the intestinal juice. In vitro digestion was performed by mixing the formulation and intestinal juice at a 1:1 ratio, followed by incubation at 37 °C for 90 min, according to Calabuig-Jiménez *et al*. [25]. After digestion, strain viability was evaluated as described for gastric digestion.

#### 2.6.3. Sequential gastric and intestinal juice simulated digestion.

For sequential digestion, a 1:1 mixture of formulation and gastric juice was incubated at 37 °C for 90 min. After incubation, the samples were centrifuged, and the pellet was resuspended in 1 mL of intestinal juice and incubated at 37 °C for an additional 90 min. Following this sequential digestion, the samples were centrifuged again, and strain viability was determined as described previously.

#### 2.6.4. Survival.

The survival of *L. plantarum* S180.7 and *L. plantarum* SBR64.7 under the different simulated digestion conditions was calculated using the following Equation 2:


$$\begin{array}{c} \text{Survival }(\%)=\frac{{\text{log}\text{N}}_{\text{1}}}{{\text{log}\text{N}}_{\text{0}}}*\text{100} \end{array}$$ (2)

Where:

- N_1_ = represents the CFU/mL at the end of the simulated digestion;
- N_0_ = represents the CFU/mL before digestion.

### 2.7. Statistical analysis

This study was conducted using a completely randomized design with four treatments (CF 1, TF1, CF2 and TF2), three repetitions in a split-plot scheme in time. The effect of time (0–24 h for fermentation and 0–28 days for storage) was considered as a repeated measure. Six different measurements of time intervals (hours) and five different measurements of storage time intervals (days) were used for growth kinetics (except for chemical analysis and survival under *in vitro* gastrointestinal conditions). The data were analyzed by fitting mixed linear models [26], with the Mixed procedure of the SAS statistical program (Sas Studio). The effects of treatments, fermentation time, and storage time were considered as fixed factors, while plot and subplot errors were treated as random effects. Variance components were estimated using the restricted maximum likelihood (REML) method. Akaike’s information criterion (AIC) was used to select the most appropriate covariance structure for the residuals [27]. Means were estimated using the LSMEANS function, and comparisons among treatments, fermentation time, storage time, or their interactions were performed using Tukey’s test at a 5% significance level. When the effect of fermentation time or storage time was significant, simple linear and quadratic regression models were adjusted using the SigmaPlot software version 14.0.

## 3. Results and discussions

### 3.1. Chemical composition

The addition of chickpea flour to milk-based formulations reduced moisture (TF1: 80.84 ± 0.05%; TF2: 80.24 ± 0.01%) and lipid content (TF1: 0.85 ± 0.07%; TF2: 1.37 ± 0.10%) while increasing carbohydrate (TF1: 14.13 ± 0.44%; TF2: 14.48 ± 0.61%) and ash concentrations (TF1: 0.87 ± 0.01%; TF2: 0.89 ± 0.02%), regardless of the strain used (*L. plantarum* S180.7 or *L. plantarum* SBR64.7), compared to their respective control formulations (CF1: 83.19 ± 0.02%; CF2: 83.04 ± 0.09%; CF1: 2.26 ± 0.04%; CF2: 1.95 ± 0.27%; CF1: 10.46 ± 0.58%; CF2: 11.40 ± 0.23%; CF1: 0.75 ± 0.01%; CF2: 0.77 ± 0.01%, respectively; Table 1). These results were expected because chickpea flour contains higher concentrations of carbohydrates (53%) and ash (7%) and lower lipid content (7%) compared to skimmed milk (39%, 0.38%, and 27%, respectively) [28].

**Table 1:** Chemical composition of skimmed milk-based formulations with *L. plantarum* S180.7 or *L. plantarum* SBR 64.7 and added or not of chickpea flour.

Formulations	Variables (%)				
	Moisture	Proteins	Lipids	Carbohydrates	Ash
**CF1**	83.19 ± 0.02^a*^	3.34 ± 0.56^a^	2.26 ± 0.04^a*^	10.46 ± 0.58^b^	0.75 ± 0.01^b^
**TF1**	80.84 ± 0.05^b*^	3.29 ± 0.47^a^	0.85 ± 0.07^b^	14.13 ± 0.44^a^	0.87 ± 0.01^a^
**CF2**	83.04 ± 0.09^a^	2.84 ± 0.01^a^	1.95 ± 0.27^a^	11.40 ± 0.23^b*^	0.77 ± 0.01^b^
**TF2**	80.24 ± 0.01^b^	3.02 ± 0.61^a^	1.37 ± 0.10^b*^	14.48 ± 0.61^a^	0.89 ± 0.02^a^

* Differences between the bacteria strains within the same formulation (CF1 *versus* CF2; TF1 *versus* TF2). Different lowercase letters indicate differences between the treatment and its control formulation according to the Tukey test with 5% probability. (CF1 *versus* TF1; CF2 *versus* TF2) at the same time. CF1 = reconstituted skimmed milk (10%) + sucrose (10%) + *L. plantarum* S180.7, TF1 = reconstituted skimmed milk (10%) + sucrose (10%) + *L. plantarum* S180.7 + 4.5% chickpea flour, CF2 = reconstituted skimmed milk (10%) + sucrose (10%) + *L. plantarum* SBR 64.7, TF2 = reconstituted skimmed milk (10%) + sucrose (10%) + *L. plantarum* SBR 64.7 + 4.5% chickpea flour.

In addition, chickpea flour has lower moisture content (4.7%) than 10% reconstituted skimmed milk, which contributed to the reduced moisture in the test formulations. Ramírez-Sucre [29] reported similar results for yogurt formulated with Mexican caramel jelly, where the control yogurt showed the highest moisture content (90.7 g/100 g) and jelly-supplemented yogurts ranged from 80.6 to 85.1 g/100 g. This confirms that higher solids content reduces moisture. Additionally, probiotic strain variations also significantly affect moisture content. Shabbir *et al*. [30] evaluated a fermented skimmed milk beverage supplemented with two probiotic strains, *Lacticaseibacillus casei* (Lc) and *Lacticaseibacillus rhamnosus* (Lr) at five concentrations (1–3%). The Lr strain yielded higher moisture (84.24 - 88.75%) than the Lc strain (84.25% to 85.65%), indicating that strain modulates the final moisture content of fermented product.

### 3.2. Growth kinetics of *L. plantarum* S180.7 and *L. plantarum* SBR64.7 strains in different formulations during the 24-hour fermentation period

#### 3.2.1. pH, titratable acidity, total soluble solids and viability of *L. plantarum* S180.7 and *L. plantarum* SBR64.7 during the 24-hour fermentation period.

The addition of chickpea flour to the milk-based formulation fermented with *L. plantarum* S180.7 (TF1) resulted in a pH reduction starting at 9 h of fermentation compared to the control (CF1). In contrast, the formulation fermented with *L. plantarum* SBR64.7 (TF2) exhibited a pH reduction from 6 h compared to its control (CF2; Fig 1A and Fig 1B). Formulations fermented with *L. plantarum* SBR64.7 (CF2 and TF2) showed lower pH values at 0, 3, and 6 h than those fermented with *L. plantarum* S180.7 (CF1 and TF1). However, at 24 h, the chickpea flour formulation fermented with *L. plantarum* S180.7 (TF1) displayed a lower pH than TF2.

**Fig 1:**
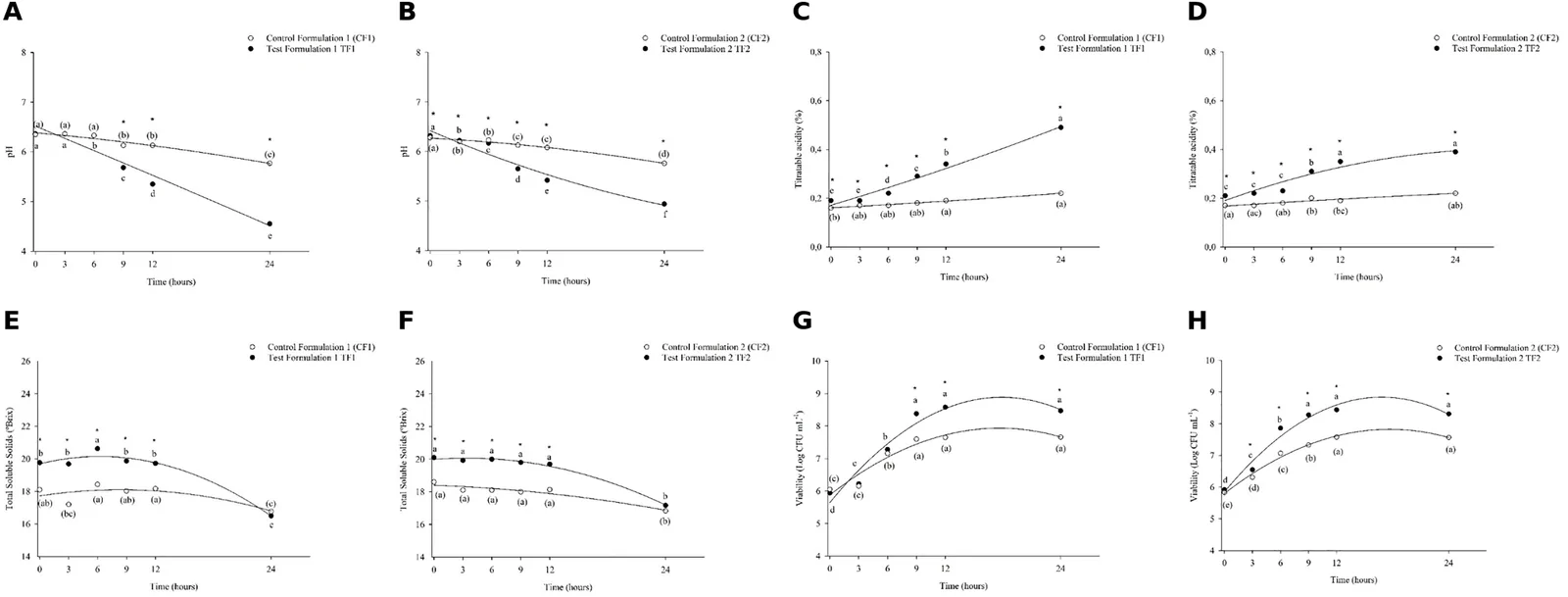
pH (A and B), titratable acidity (C and D), total soluble solids (E and F) and viability (G and H) of *L. plantarum* S180.7 and *L. plantarum* SBR64.7 in skimmed milk-based formulations added or not of chickpea flour, during 24 h of fermentation at 37°C. * Differences between test and control formulations within the same bacteria strain (CF1 *versus* TF1; CF2 *versus* TF2). Different lowercase letters indicate differences during the fermentation period within the same formulation. According to the Tukey test with 5% probability. CF1 = reconstituted skimmed milk (10%) + sucrose (10%) + *L. plantarum* S180.7, TF1 = reconstituted skimmed milk (10%) + sucrose (10%) + *L. plantarum* S180.7 + 4.5% chickpea flour; CF2 = reconstituted skimmed milk (10%) + sucrose (10%) + *L. plantarum* SBR 64.7, TF2 = reconstituted skimmed milk (10%) + sucrose (10%) + *L. plantarum* SBR 64.7 + 4.5% chickpea flour.

During fermentation period, the formulations fermented with *L. plantarum* SBR64.7 (CF2 and TF2) showed a pH decrease starting at 3 h compared to time 0, whereas formulations with *L. plantarum* S180.7 (CF1 and TF1) exhibited pH reductions only after 9 h and 6 h of fermentation, respectively. Regarding titratable acidity inversely proportional results were found (Fig 1C and Fig 1D). Formulations supplemented with chickpea flour (TF1 and TF2) exhibited higher titratable acidity than their respective controls (CF1 and CF2), regardless of fermentation time or bacterial strain. Among the controls, the formulation fermented with *L. plantarum* SBR64.7 (CF2) showed higher titratable acidity at 0 and 9 h of fermentation compared to the formulation fermented with *L. plantarum* S180.7 (CF1). A similar pattern was observed among the formulations containing chickpea flour, with TF2 exhibiting higher titratable acidity than TF1 at 0, 3, and 9 h of fermentation. However, after 24 h of fermentation, titratable acidity was higher in TF1 compared to TF2. During fermentation, the formulations without chickpea flour (CF1 and CF2) showed increases in titratable acidity starting at 12 h and 9 h, respectively, relative to time 0. In the formulations containing chickpea flour, increases in titratable acidity were observed from 6 h for TF1 and 9 h for TF2.

Total soluble solids (TSS, °Brix) were higher in the formulations added with chickpea flour (TF1 and TF2) compared to their respective controls (CF1 and CF2) up to 12 h of fermentation (Fig 1E and Fig 1F). At 6 h of fermentation, the TSS value was higher in the TF1 formulation compared to TF2. Across the fermentation period, a decrease in TSS was observed in all formulations only at 24 h, relative to 0 h. A decrease in pH was also observed in the formulations containing chickpea flour (TF1 and TF2) compared to their respective controls. Organic acids are the main metabolites produced by lactic acid bacteria, leading to a decrease in pH during the fermentation process [31]. Although both strains demonstrated the ability to ferment chickpea flour components, *L. plantarum* SBR64.7 appeared to be more efficient, as the TF2 formulation showed a faster pH decrease at 3 h of fermentation, whereas in the formulation with *L. plantarum* S180.7, the pH decrease was observed only after 6 h. However, when considering the pH decrease at the end of the fermentation period, the formulation fermented with *L. plantarum* S180.7 and added with chickpea flour showed a greater reduction (ΔpH{24–0h}: TF1 = –1.81) compared to the formulation fermented with *L. plantarum* SBR64.7 and added with chickpea flour (ΔpH{24–0h}: TF2 = –1.38). This result suggests a better long-term adaptation of the *L. plantarum* S180.7 strain to a medium containing chickpea flour.

Similarly to our results, Coman *et al*. [32] reported that the supplementation of fermented milk with buckwheat flour and oat bran enhanced the acidifying ability of *Lactobacillus paracasei* IMC 502® compared to the control after 4 weeks of storage at 4 °C. Total soluble solids (TSS) consist primarily of low-molecular-weight carbohydrates, particularly mono- and disaccharides, that are readily soluble in aqueous media. These compounds serve as fermentable substrates for lactic acid bacteria, supporting glycolytic flux and subsequent organic acid production, which directly influences microbial growth kinetics and acidification capacity during fermentation. The higher amount of total soluble solids (TSS) observed in the formulations added with chickpea flour (TF1 and TF2) may be attributed to its high content of oligosaccharides, such as raffinose and ciceritol. The greater reduction in TSS values observed in TF1 and TF2 (ΔTSS{24–0h} = –3.27 and –2.93, respectively) compared to CF1 and CF2 (ΔTSS{24–0h} = –1.33 and –1.77, respectively) at the end of the fermentation period indicates that both strains, *L. plantarum* S180.7 and *L. plantarum* SBR64.7, were able to metabolize the complex carbohydrates present in chickpea flour. During fermentation, lactic acid bacteria (LAB) hydrolyze carbohydrates into soluble organic acids, leading to a decrease in TSS values [33,34]. Similar results were reported by Norberto *et al*. [35], who observed a reduction in TSS in a soy-based beverage fermented with kefir grains throughout the fermentation process.

The addition of chickpea flour increased the population of *L. plantarum* S180.7 and *L. plantarum* SBR64.7 in milk-based formulations (Fig 1G and Fig 1H). For the *L. plantarum* S180.7 strain, the increase in viability was observed only after 9 h of fermentation (CF1 *versus* TF1), whereas for *L. plantarum* SBR64.7 (CF2 *versus* TF2), the increase occurred from 3 h. Throughout the fermentation period, both strains exhibited a similar growth profile, with viability increasing up to 9 h, followed by stabilization up to 24 h, regardless of the formulation. In the control formulations, *L. plantarum* S180.7 exhibited higher viability at 0 and 9 h of fermentation compared to *L. plantarum* SBR64.7. In the test formulations, *L. plantarum* SBR64.7 showed greater viability at 3 and 6 h compared to *L. plantarum* S180.7.

In the control formulations, *L. plantarum* S180.7 exhibited higher viability at 0 and 9 h of fermentation compared to *L. plantarum* SBR64.7. In the test formulations, *L. plantarum* SBR64.7 showed greater viability at 3 and 6 h compared to *L. plantarum* S180.7. Both *L. plantarum* S180.7 and *L. plantarum* SBR64.7 maintained viability above the minimum level required for a food to be considered probiotic (6–8 log CFU/mL) in all formulations [36]. However, in formulations supplemented with 4.5% chickpea flour, the strains exhibited higher viability, reaching 8 log CFU/mL. A similar prebiotic effect was reported by Chen *et al*. [37], where chickpea flour stimulated the growth and maintained higher counts of *L. delbrueckii* ssp. *bulgaricus* in yogurt, suggesting that minerals, such as zinc, and oligosaccharides present in chickpea flour enhanced the activity of the bacterial cultures. Chickpea matrix is characterized by a high content of carbohydrates (63.0 g/100 g) and dietary fiber (12.2 g/100 g). Starch is the main carbohydrate present, while free sugars (glucose and fructose), disaccharides (sucrose), and oligosaccharides (raffinose) are also present. Additionally, chickpeas fiber profile includes soluble fraction (pectins and gums), insoluble fraction (cellulose, hemicellulose, and lignin) and resistant starch. All these compounds contribute to increasing the viability of lactic acid bacteria, as they provide energy, exert prebiotic effects, and improve bacterial stability during storage [38,39].

Among the nutrients that enhance the viability of *Lactobacillus plantarum* strains (*L. plantarum* ST-III, WCFS1, and LPHS) during fermentation in dairy matrices are amino acids [7]. Chickpea proteins provide a well-balanced profile of amino acids, including glutamic acid, aspartic acid, arginine, leucine, phenylalanine, lysine, and serine. Thus, the amino acids and carbohydrates in chickpea flour serve as substrates for fermentation [40], which may explain the higher viability of both strains in formulations supplemented with chickpea flour.

#### 3.2.2. Total phenolics content and the antioxidant potential of formulations.

Formulations added with chickpea flour (TF1 and TF2) exhibited higher total phenolic content at all fermentation times compared to their respective controls (CF1 and CF2), regardless of the strain used (Fig 2A and Fig 2B). Among the chickpea-supplemented formulations, TF2 (*L. plantarum* SBR64.7) showed higher total phenolic content at 0 and 3 h compared to TF1 (*L. plantarum* S180.7). During fermentation, only the chickpea-supplemented formulations (TF1 and TF2) showed a reduction in total phenolics from 9 h and 6 h, respectively, after which the values remained stable until 24 h.

**Fig 2:**
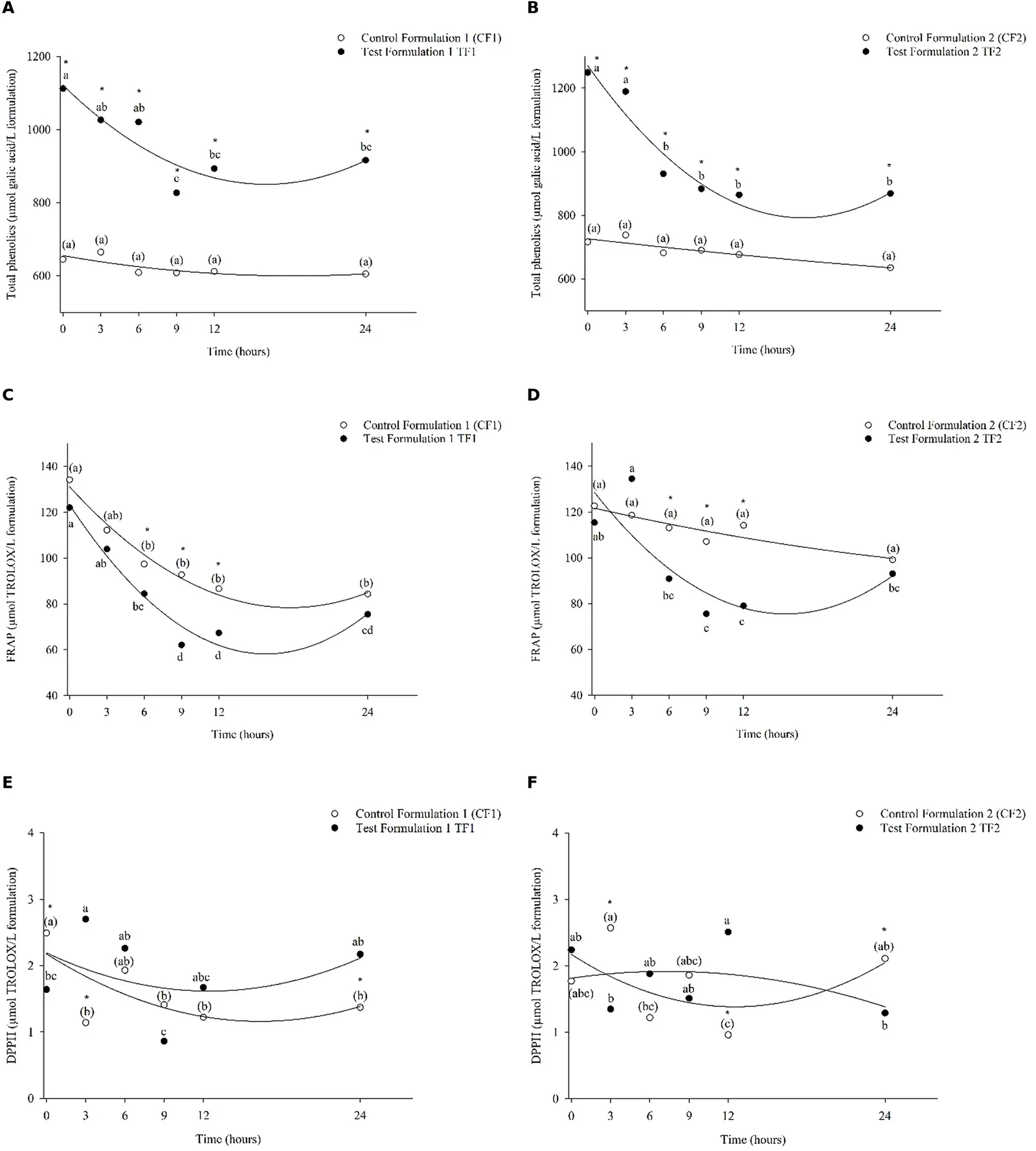
Total phenolics (A and B), FRAP (C and D) and DPPH (E and F) of *L. plantarum* S180.7 and *L. plantarum* SBR64.7 in skimmed milk-based formulations added or not of chickpea flour, during 24 h of fermentation at 37°C. * Differences between test and control formulations within the same bacteria strain (CF1 *versus* TF1; CF2 *versus* TF2). Different lowercase letters indicate differences during the fermentation period within the same formulation. According to the Tukey test with 5% probability. CF1 = reconstituted skimmed milk (10%) + sucrose (10%) + *L. plantarum* S180.7, TF1 = reconstituted skimmed milk (10%) + sucrose (10%) + *L. plantarum* S180.7 + 4.5% chickpea flour; CF2 = reconstituted skimmed milk (10%) + sucrose (10%) + *L. plantarum* SBR 64.7, TF2 = reconstituted skimmed milk (10%) + sucrose (10%) + *L. plantarum* SBR 64.7 + 4.5% chickpea flour.

Regarding the antioxidant potential assessed by the FRAP assay, the TF1 and TF2 formulations exhibited a reduction in FRAP values between 6 and 12 h of fermentation compared to their respective controls (CF1 and CF2). At 3 and 24 h, the FRAP value was higher in TF2 than in TF1, whereas at 6 and 12 h, CF2 showed higher FRAP values than CF1. Over the 24 h fermentation period, CF1 displayed a decrease in FRAP at 6 h followed by stabilization until 24 h, while CF2 remained stable throughout. Both TF1 and TF2 formulations showed a reduction in FRAP after 6 h, remaining stable from 9 to 24 h.

The antioxidant capacity estimated by the reduction of the DPPH^●^ free radical at the end of 24 h of fermentation was higher in the formulation added with chickpea flour and fermented with *L. plantarum* S180.7 (TF1) compared to its control (CF1). In contrast, the TF2 formulation (*L. plantarum* SBR64.7 with chickpea flour) showed a reduction in antioxidant capacity relative to CF2. Over the fermentation period, CF1 exhibited a decrease in DPPH● values from 9 h compared to 0 h, whereas CF2 remained stable. In the chickpea-supplemented formulations (TF1 and TF2), no significant changes in DPPH● values were observed after 24 h compared to 0 h.

The higher total phenolic content in the chickpea-supplemented formulations (TF1 and TF2) is consistent with the composition of chickpeas, which contain various phytochemicals, including phenolic compounds [34,40]. Hussein *et al*. [33] also reported that yogurt supplemented with chickpea flour exhibited higher phenolic content and antioxidant activity compared to the control. Similar to our findings, where total phenolic content decreased during fermentation in the chickpea-supplemented formulations, Zhu *et al*. [34] observed a reduction in total phenolic content in chickpea milk fermented with different *Lactobacillus* strains. However, while Zhu *et al*. [34] reported this reduction after 20 h of fermentation, in the present study it occurred after 6 h. The authors attributed the decrease in phenolic content to condensation with proteins and subsequent precipitation, or to metabolites produced by *Lactobacillus* that reduce phenolic content to facilitate bacterial growth.

Therefore, the reduction in antioxidant potential, as estimated by the FRAP assay, in the chickpea-supplemented formulations during fermentation may be related to the decrease in phenolic compound concentration, which are major contributors to antioxidant activity (Fig 2C and Fig 2D). The apparent discrepancy between the antioxidant potential of TF1 and TF2 formulations measured by DPPH● and FRAP assays during fermentation can be attributed to differences in the reactivity of antioxidants with the assay reagents (Fig 2E and Fig 2F). The FRAP assay measures the reduction of ferric to ferrous ions by electron-donating antioxidants, whereas the DPPH● assay evaluates the ability of antioxidants to scavenge the 1,1-diphenyl-2-picrylhydrazyl free radical [41].

### 3.3. Physicochemical and microbiological characterization of formulations fermented for 24 hours and stored for 28 days at 4°C

#### 3.3.1. pH, titratable acidity, total soluble solids and viability of *L. plantarum* S180.7 and *L. plantarum* SBR64.7 of formulations fermented for 24 hours and stored for 28 days at 4°C.

All formulations showed a reduction in pH values and an increase in total titratable acidity throughout the 28 days of storage. However, in the formulations containing chickpea flour (TF1 and TF2), the reduction in pH was 2.8- and 2.3-fold higher compared to the control formulations (ΔpH_(28–0 d)_: CF1 = –0.5 vs. TF1 = –1.4; CF2 = –0.5 vs. TF2 = –1.08), accompanied by an approximately fourfold increase in total titratable acidity _(28–0 d)_: CF1 = 0.07 vs. TF1 = 0.30; CF2 = 0.06 vs. TF2 = 0.24; Fig 3C and Fig 3D). The decrease in pH was observed after 7 days of storage in all formulations. However, in the control formulations (CF1 and CF2), the increase in titratable acidity was only detected after 14 days at 4 °C, whereas in the formulations with chickpea flour (TF1 and TF2), it occurred earlier, at 7 days.

**Fig 3:**
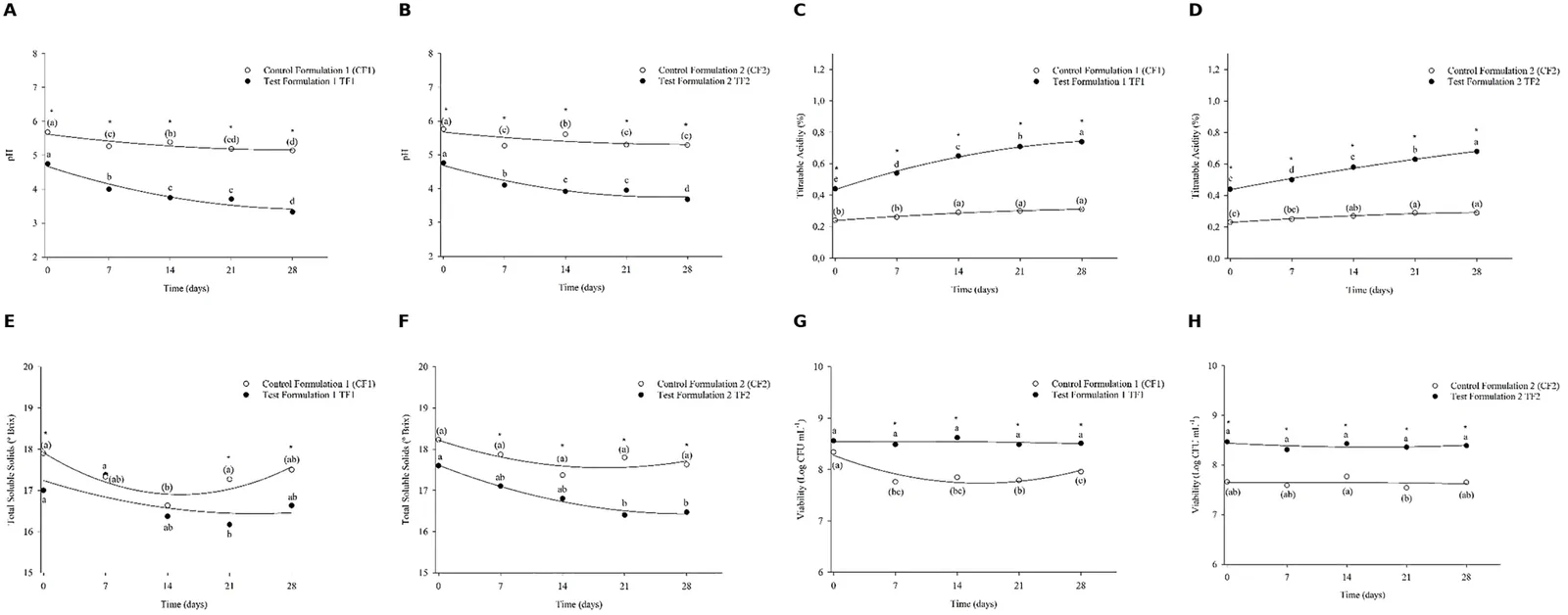
pH (A and B), titratable acidity (C and D), total soluble solids (E and F) and viability (G and H) of *L. plantarum* S180.7 and *L. plantarum* SBR64.7 in skimmed milk-based formulations added or not of chickpea flour, during 28 days of storage at 4°C. * Differences between test and control formulations within the same bacteria strain (CF1 *versus* TF1; CF2 *versus* TF2). Different lowercase letters indicate differences during the fermentation period within the same formulation. According to the Tukey test with 5% probability. CF1 = reconstituted skimmed milk (10%) + sucrose (10%) + *L. plantarum* S180.7, TF1 = reconstituted skimmed milk (10%) + sucrose (10%) + *L. plantarum* S180.7 + 4.5% chickpea flour; CF2 = reconstituted skimmed milk (10%) + sucrose (10%) + *L. plantarum* SBR 64.7, TF2 = reconstituted skimmed milk (10%) + sucrose (10%) + *L. plantarum* SBR 64.7 + 4.5% chickpea flour.

The control formulation containing *L. plantarum* S180.7 (CF1) showed lower pH values than the control with *L. plantarum* SBR64.7 (CF2) at 0, 14, 21, and 28 days of storage at 4 °C. A similar trend was observed in the formulations with chickpea flour, in which TF1 presented lower pH values than TF2; however, this difference was evident only after 7 days of storage (Fig 3A and Fig 3B).

Regarding total soluble solids (TSS, °Brix), the TF1 and TF2 formulations showed lower values compared to their respective controls (CF1 and CF2) (Fig 3E and Fig 3F). In the formulations with *L. plantarum* S180.7 (CF1 and TF1), this difference was detected at 21 and 28 days of storage, whereas in the formulations with *L. plantarum* SBR64.7 (CF2 and TF2), it was evident earlier, after 7 days. Throughout storage at 4 °C, only the formulations containing chickpea flour exhibited a reduction in TSS. In TF1, this reduction was observed only at 21 days, while in TF2 it occurred at both 21 and 28 days.

Taken together, these results suggest that the fermentative activity observed at low temperature may be associated with the increased carbohydrate content provided by the addition of chickpea flour. Moreover, *L. plantarum* S180.7 appears to be more efficient in metabolizing the chickpea matrix at low storage temperatures, as the TF1 formulation exhibited lower pH and higher titratable acidity than TF2, which was fermented by *L. plantarum* SBR64.7, during the 28 days of storage. Although high acidity may reduce product acceptability, some yoghurts with pH values as low as 3.7–3.8, such as Greek-style yoghurt, are still well accepted by consumers [42]. Similarly, Hussein *et al*. [33] reported that a yoghurt supplemented with chickpea flour and fermented with *Bifidobacterium bifidum* or with a mixture of *Bifidobacterium animalis* subsp. *lactis* and *Lactobacillus acidophilus* showed a decrease in pH and an increase in titratable acidity compared to the control bio-yoghurt without chickpea flour during 21 days of cold storage.

The addition of chickpea flour to milk-based formulations fermented for 24 h enhanced the viability of both *L. plantarum* S180.7 and *L. plantarum* SBR64.7, as the colony-forming unit counts at 7, 14, 21, and 28 days of storage at 4 °C were higher in TF1 and TF2 compared to their respective controls, CF1 and CF2 (Fig 3G and 3H). During storage at 4 °C, the viability of *L. plantarum* S180.7 in the control formulation (CF1) decreased after 7 days and then remained constant up to 28 days, whereas *L. plantarum* SBR64.7 in CF2 maintained stable viability throughout storage. In the formulations supplemented with chickpea flour, both strains (*L. plantarum* S180.7 and *L. plantarum* SBR64.7) retained stable viability during the entire storage period.

At 0, 21, and 28 days of storage, *L. plantarum* S180.7 exhibited higher viability than *L. plantarum* SBR64.7 in the control formulations. Similarly, among the test formulations, *L. plantarum* S180.7 showed greater viability than *L. plantarum* SBR64.7 at 28 days of storage.

It is important to note that even at pH values below 4, the counts of *L. plantarum* S180.7 and *L. plantarum* SBR64.7 remained above 8 log CFU/mL in the TF1 and TF2 formulations during 28 days of storage at 4 °C, exceeding the minimum level of 7 log CFU/mL required by legislation for live cultures throughout shelf life [43]. The presence of oligosaccharides, free amino acids, and phenolic compounds in chickpeas may explain the high viability of the strains under low-pH conditions during refrigerated storage [33], as these components of chickpea flour could serve as substrates for growth and maintenance of the strains in the formulations.

#### 3.3.2. Visual stability and water retention capacity.

The formulations supplemented with chickpea flour (TF1 and TF2) showed lower water release values (greater stability) and, consequently, higher water retention capacity than their respective controls (CF1 and CF2) at both 0 and 28 days of storage at 4 °C (Table 2), regardless of the strain used (*L. plantarum* S180.7 or *L. plantarum* SBR64.7. These results indicate that TF1 and TF2 remained more stable during storage.

**Table 2:** Visual stability and water holding capacity of skimmed milk-based formulations with *L. plantarum* S180.7 or *L. plantarum SBR 64.7* and added or not of chickpea flour, during 28 days of storage at 4°C.

Formulations	Stability (mL)		Water holding capacity (%)	
	Storage time (days)			
	0	28	0	28
**CF1**	0.29 ± 0.05^a;B*^	0.58 ± 0.05^a;A^	4.06 ± 2.35^b;A^	3.57 ± 1.34^b;A^
**TF1**	0.09 ± 0.00^b;A^	0.12 ± 0.05^b;A^	27.50 ± 4.53^a;B^	57.89 ± 1.88^a;A*^
**CF2**	0.18 ± 0.00^a;B^	0.58 ± 0.05^a;A^	7.74 ± 1.53^b;A^	2.10 ± 0.44^b;A^
**TF2**	0.09 ± 0.00^b;B^	0.27 ± 0.00^b;A*^	20.63 ± 8.21^a;B^	50.15 ± 4.19^a;A^

* Differences between the bacteria strains within the same formulation (CF1 *versus* CF2; TF1 *versus* TF2). Different lowercase letters indicate differences between the treatment and its control formulation (CF1 *versus* TF1; CF2 *versus* TF2) at the same time. Different capital letters indicate differences throughout fermentation within the formulation according to the Tukey test with 5% probability. CF1 = reconstituted skimmed milk (10%) + sucrose (10%) + *L. plantarum* S180.7, TF1 = reconstituted skimmed milk (10%) + sucrose (10%) + *L. plantarum* S180.7 + 4.5% chickpea flour, CF2 = reconstituted skimmed milk (10%) + sucrose (10%) + *L. plantarum* SBR 64.7, TF2 = reconstituted skimmed milk (10%) + sucrose (10%) + *L. plantarum* SBR 64.7 + 4.5% chickpea flour.

With respect to storage time, the control formulations (CF1 and CF2) exhibited higher water release values at 28 days compared to day 0. Among the test formulations, only TF2 showed an increase in water release between days 0 and 28, whereas no difference was observed in TF1. At day 28, the water retention capacity of TF1 and TF2 was 2.1- and 2.4-fold higher, respectively, compared to day 0, while no difference was observed in the control formulations over the same period.

Polysaccharides and oligosaccharides are known to retain water molecules, acting as natural thickening and gelling agents (33). Thus, chickpea polysaccharides may have contributed to the improved stability of milk-based formulations by enhancing water retention capacity. The reduction of syneresis observed during refrigerated storage in formulations containing chickpea flour supports this, indicating that chickpea flour improves the stability of milk-based products over 28 days. Similar findings were reported by Vogado *et al*. [44], who showed that green banana pulp reduced syneresis in fermented milk due to its high carbohydrate content.

#### 3.3.3. Total phenolic content and antioxidant capacity of formulations after 28 days of storage at 4°C.

The concentration of total phenolics remained constant during the 28 days of storage at 4 °C in all formulations. However, the antioxidant potential estimated by the FRAP and DPPH● assays remained stable only in the formulations supplemented with chickpea flour. In contrast, the control formulations showed a decrease in antioxidant potential after 28 days of storage, regardless of the strain used, *L. plantarum* S180.7 or *L. plantarum* SBR64.7 (Table 3).

**Table 3:** Total phenolics content and the antioxidant capacity of skimmed milk-based formulations with *L. plantarum* S180.7 or *L. plantarum* SBR 64.7 and added or not of chickpea flour, during 28 days of storage at 4°C.

	Formulations	Storage time (days)	
		0	28
**TOTAL PHENOLICS** **(µmol Gallic Acid/L formulation)**	**CF1**	809.1 ± 25.6^b;A^	718.1 ± 88.3^b;A^
	**TF1**	1171.8 ± 184.2^a;A^	1118.5 ± 97.0^a;A^
	**CF2**	828.5 ± 52.1^b;A^	850.7 ± 56.5^b;A^
	**TF2**	1274.0 ± 100.2^a;A^	1202.9 ± 56.4^a;A^
**FRAP** **(µmol TROLOX/L formulation)**	**CF1**	120.5 ± 11.3^a;A^	82.7 ± 11.6^a;B^
	**TF1**	102.4 ± 8.6^b;A^	93.6 ± 6.6^a;A^
	**CF2**	137.6 ± 6.4^a;A*^	108.0 ± 4.8^a;B*^
	**TF2**	114.8 ± 6.5^b;A^	102.3 ± 0.9^a;A^
**DPPH** **(µmol TROLOX/L formulation)**	**CF1**	1.43 ± 0.27^a;B^	2.83 ± 0.42^a;A*^
	**TF1**	1.76 ± 0.51^a;A^	1.49 ± 0.30^b;A^
	**CF2**	2.52 ± 0.12^a;A*^	2.09 ± 0.20^a;B^
	**TF2**	1.36 ± 0.16^b;A^	1.54 ± 0.54^a;A^

* Differences between the bacteria strains within the same formulation (CF1 *versus* CF2; TF1 *versus* TF2). Different lowercase letters indicate differences between the treatment and its control formulation (CF1 *versus* TF1; CF2 *versus* TF2) at the same time. Different capital letters indicate differences throughout fermentation within the formulation according to the Tukey test with 5% probability. CF1 = reconstituted skimmed milk (10%) + sucrose (10%) + *L. plantarum* S180.7, TF1 = reconstituted skimmed milk (10%) + sucrose (10%) + *L. plantarum* S180.7 + 4.5% chickpea flour, CF2 = reconstituted skimmed milk (10%) + sucrose (10%) + *L. plantarum* SBR 64.7, TF2 = reconstituted skimmed milk (10%) + sucrose (10%) + *L. plantarum* SBR 64.7 + 4.5% chickpea flour.

Contrary to our findings, the storage of a yoghurt added with chickpea flour (1, 2, and 3%) for 21 days at 4 °C and fermented by *Bifidobacterium bifidum* or a mixture of *Bifidobacterium animalis* subsp. *lactis* and *Lactobacillus acidophilus* resulted in a reduction of total phenolics and antioxidant capacity. The authors attributed this decrease to the presence of oxygen and the consequent degradation of certain phenolic compounds. The discrepancies between studies may be explained by the different percentages of chickpea flour used (1–3% *versus* 4.5%) and by the distinct matrices, namely yogurt versus 10% reconstituted milk [33].

Although the formulations supplemented with chickpea flour showed a higher concentration of total phenolics, their FRAP values at day 0 of storage were lower (TF1: 102.42 ± 8.63 and TF2: 114.78 ± 6.49 µmol Trolox/L) compared with the control formulations CF1 and CF2 (120.49 ± 11.31 and 137.63 ± 6.35 µmol Trolox/L, respectively). This outcome may be related to the distinct antioxidant mechanisms of phenolic compounds, since some act primarily as radical scavengers, while others function as reducing agents or metal chelators.

### 3.4. Survival of *L. plantarum* S180.7 and *L. plantarum* SBR64.7 in formulations after *in vitro* exposure to simulated gastrointestinal tract conditions

The viability of the *L. plantarum* S180.7 strain in the control formulation (CF1) significantly decreased after exposure to gastric juice in vitro, whereas the *L. plantarum* SBR64.7 strain showed a reduction only after sequential exposure to gastric and pancreatic juices. In contrast, the viability of both strains remained unchanged after exposure to gastric juice, pancreatic juice, or their sequential combination in the formulations supplemented with chickpea flour (Table 4). Overall, the viability of the strains, both before and after simulated in vitro digestion, was higher in the formulations with chickpea flour (TF1 and TF2) compared to the control formulations (CF1 and CF2).

**Table 4:** Viability and survival of *L. plantarum* S180.7 or *L. plantarum* SBR 64.7 in skimmed milk-based formulations added or not of chickpea flour, during gastrointestinal *in vitro* digestion.

*in vitro* simulated digestion					
	Formulations	Before	After		
			Gastric juice	Pancreatic juice	Sequential (GJ + PJ)
**Viability (Log CFU mL**^**-1**^)	**CF1**	7.60 ± 0.05^b*^	7.20 ± 0.13^b;B*^	7.32 ± 0.23^b;A^	7.30 ± 0.12^b;A^
	**TF1**	8.34 ± 0.05^a^	8.07 ± 0.10^a;A^	8.21 ± 0.04^a;A^	8.12 ± 0.02^a;A^
	**CF2**	7.49 ± 0.05^b^	7.22 ± 0.03^b;A^	7.29 ± 0.06^b;A^	7.20 ± 0.18^b;B^
	**TF2**	8.16 ± 0.10^a^	8.07 ± 0.06^a;A^	8.08 ± 0.07^a;A^	8.03 ± 0.01^a;A^
**Survival (%)**	**CF1**	100	94.70 ± 2.10^a;A^	96.23 ± 2.91^a;A^	96.05 ± 1.40^a;A^
	**TF1**	100	96.80 ± 0.79^a;A^	98.45 ± 1.06^a;A^	97.32 ± 0.44^a;A^
	**CF2**	100	96.44 ± 0.93^a;A^	97.29 ± 0.65^a;A^	96.13 ± 2.49^a;A^
	**TF2**	100	98.90 ± 0.85^a;A^	98.98 ± 0.57^a;A^	98.33 ± 1.16^a;A^

* Differences between viability before exposure to juices and after *in vitro* simulated digestion within the same formulation. Different lowercase letters indicate differences between the treatment and its control formulation (CF1 *versus* TF1; CF2 *versus* TF2) at the same time. Different capital letters indicate differences throughout *in vitro* simulated digestion within the formulation according to the Tukey test with 5% probability. CF1 = reconstituted skimmed milk (10%) + sucrose (10%) + *L. plantarum* S180.7, TF1 = reconstituted skimmed milk (10%) + sucrose (10%) + *L. plantarum* S180.7 + 4.5% chickpea flour, CF2 = reconstituted skimmed milk (10%) + sucrose (10%) + *L. plantarum* SBR 64.7, TF2 = reconstituted skimmed milk (10%) + sucrose (10%) + *L. plantarum* SBR 64.7 + 4.5% chickpea flour.

These results demonstrate that the addition of chickpea flour to a milk-based matrix protected both *L. plantarum* SBR64.7 and *L. plantarum* S180.7 against gastric, pancreatic, and sequential stress conditions, maintaining their viability at levels similar to those observed prior to exposure. Leandro *et al*. [4], when isolating these potentially probiotic strains, reported only a 1.16 and 1.24 logarithmic cycle reduction in viability after pancreatic exposure, respectively. Thus, these strains already exhibited intrinsic resistance to this type of stress, which, based on our findings, can be further enhanced by the food matrix. Components of chickpea flour may interact with bile salts, thereby protecting the bacterial membrane from their toxic effects, while other compounds may directly shield bacterial cells under harsh conditions. Furthermore, carbohydrates are the most abundant compounds in chickpeas, including oligosaccharides such as raffinose, and polysaccharides such as starch, stachyose, and verbascose. These molecules are particularly relevant because they can act as a physical barrier, offering protection against fatty acids and bile salts [45].

In agreement with our findings, the addition of apple, banana, and grape flours (1%) to fermented milk was reported to exert a protective effect on the simulated gastrointestinal resistance of *Bifidobacterium animalis* subsp. *lactis* BB-12 after 28 days of storage [46].

## 4. Conclusion

In summary, the incorporation of chickpea flour into milk-based formulations enhanced the viability and stability of *L. plantarum* S180.7 and *L. plantarum* SBR64.7 during refrigerated storage, while also improving the functional properties of the product by increasing phenolic content and maintaining antioxidant potential. The formulations added with chickpea flour reached the minimum viable counts required for a probiotic food, highlighting the potential of this matrix to support the probiotic capacity of both strains. Notably, chickpea flour appeared to provide greater support for *L. plantarum* S180.7, suggesting a strain-dependent response. These findings demonstrate that chickpea flour is a promising functional ingredient for the development of probiotic dairy alternatives; however, further *in vivo* studies are required to confirm its efficacy and health benefits.
